# Exocomets size distribution in the $\beta$ Pictoris planetary system

**DOI:** 10.1038/s41598-022-09021-2

**Published:** 2022-04-28

**Authors:** Alain Lecavelier des Etangs, Lucie Cros, Guillaume Hébrard, Eder Martioli, Marc Duquesnoy, Matthew A. Kenworthy, Flavien Kiefer, Sylvestre Lacour, Anne-Marie Lagrange, Nadège Meunier, Alfred Vidal-Madjar

**Affiliations:** 1 Institut d’astrophysique de Paris, CNRS, UMR 7095, Sorbonne Université, 98 bis bd Arago, 75014 Paris, France; 2 Ecole nationale supérieure des mines de Paris, Université PSL, 60 boulevard Saint-Michel, 75272 Paris, France; 3 Observatoire de Haute-Provence, 04870, St Michel l’Observatoire, France; 4 Laboratório Nacional de Astrofísica, Rua Estados Unidos 154, 37504-364 Itajubá, MG Brazil; 5 LESIA, Observatoire de Paris, Université PSL, CNRS, Sorbonne Université, Univ. Paris Diderot, Sorbonne Paris Cité, 5 place Jules Janssen, 92195 Meudon, France; 6 Leiden Observatory, Leiden University, Leiden, The Netherlands; 7 Univ. Grenoble Alpes, CNRS, IPAG, 38000 Grenoble, France

**Keywords:** Astronomy and planetary science, Astronomy and astrophysics

## Abstract

The star $\beta$ Pictoris harbors a young planetary system of about 20 million years old, which is characterized by the presence of a gaseous and dusty debris disk, at least two massive planets and many minor bodies. For more than thirty years, exocomets transiting the star have been detected using spectroscopy, probing the gaseous part of the cometary comas and tails. The detection of the dusty component of the tails can be performed through photometric observations of the transits. Since 2018, the Transiting Exoplanet Survey Satellite has observed $\beta$ Pic for a total of 156 days. Here we report an analysis of the TESS photometric data set with the identification of a total of 30 transits of exocomets. Our statistical analysis shows that the number of transiting exocomet events (*N*) as a function of the absorption depth (*AD*) in the light curve follows a power law in the form $dN(AD) \propto AD^{-\alpha }$, where $\alpha =2.3\pm 0.4$. This distribution of absorption depth leads to a differential comet size distribution proportional to $R^{-\gamma }$, where $\gamma =3.6 \pm 0.8$, showing a striking similarity to the size distribution of comets in the Solar system and the distribution of a collisionally relaxed population ($\gamma _{{\text{D}}}= 3.5$).

## Introduction

Since the mid-1980’s, spectroscopic observations of the bright southern star $\beta$ Pictoris, or $\beta$ Pic, have revealed variations in the calcium H and K lines, which have been interpreted as the transit of the gaseous tails of exocomets^1–5^. Several decades of exocomets transit observations, mostly on $\beta$ Pic but also on a few other stars, provided original insight on the activity of minor bodies in planetary system within the first tens of million years^6^. For that, the $\beta$ Pic system is exceptional in presenting simultaneously several favorable properties : it is only a few tens million years old, very nearby at 19.3 parsecs and seen exactly edge-on from the Earth^7–16^. Nonetheless, even for $\beta$ Pic, the observations of exocomets were limited to the gaseous part of the tails probed by transit spectroscopy; observations of the dusty part of the tails indeed require high accuracy photometric measurements, which was until recently not available^17–21^.

The situation changed when, using data obtained by the Transiting Exoplanet Survey Satellite (TESS^21^) between October 2018 and February 2019, three photometric events were discovered^20^ and interpreted as due to the transit of the dust component of exocomets transiting $\beta$ Pic. In support of this interpretation, the observed light curves are almost identical to the predictions made twenty years earlier^17,18^.

Since 2019, $\beta$ Pic has been re-observed by TESS. Here we present the analysis of the complete data set gathered up to February 2021 in order to perform a deep search for exocomet transits and determine the size distribution of the $\beta$ Pic comets to be compared with the distribution observed in the Solar system.

## Search for exocomet transits

Considering all TESS observations of $\beta$ Pic until February 2021, after the cleaning process of the $\delta$ Scuti variations and other slower variations (see Methods) we end up with a 156 days light curve clearly showing some dips, which are similar to what is expected for exocomet transits (Extended Data Fig. 4). To make sure that the observed dips in the $\beta$ Pic light curve are real and not noise residuals nor artifacts due to the reduction process, we used the TESS observations of the nearby star $\alpha$ Pic. We downloaded the PDC flux time series of $\alpha$ Pic and applied the same procedure to remove the $\delta$ Scuti and slower variations. The observations of $\alpha$ Pic provide an excellent data-set to test our procedure because $\alpha$ Pic is in the same region of the sky as $\beta$ Pic (and has hence overlapping TESS observations epochs), it has the same spectral type (A8V versus A5V for $\beta$ Pic), similar magnitude (3.3 versus 3.85 for $\beta$ Pic) and a similarly high $v\sin i$ projected rotational velocity (205 km/s for $\alpha$ Pic versus 139 km/s for $\beta$ Pic). Thus, $\alpha$ Pic is almost a nearby stellar twin of $\beta$ Pic except for the presence of the young planetary system. $\alpha$ Pic has already been successfully used as a reference star for analysis of $\beta$ Pic observations^22^.

The $\alpha$ Pic light curve is used to check for systematics that could mimic transit of exocomets. The light curve of $\alpha$ Pic shows noisy excursions from the mean value both in the positive and negative deviations with the same pattern. The light curve of $\beta$ Pic also shows noisy excursions in the positive direction that are similar to the ones observed in the light curve of $\alpha$ Pic, but noisy excursions that are more pronounced and more frequent in the negative direction. The latest are typical signatures of small, transiting objects with extended dust tails. We do not detect similar variations in $\alpha$ Pic that the ones seen in $\beta$ Pic that can be due to the passage of exocomets in front of the star.

To characterize this excess of transit-like features in the light curve of $\beta$ Pic and identify the corresponding individual events, we calculated the correlation of the light curves with a simple model of an exocomet transit photometric event, assuming a 1D transit of a translucent dust cloud with an exponential decrease of the optical thickness from the head of the comet. This model has only four parameters : *K*, the cloud optical thickness at the leading head, $\Delta t$, the transit duration corresponding to the time needed to cover the chord length of the stellar disk at the transit velocity, $\beta$, the speed of the transit of one scale length of the cometary tail (the inverse of the scale length of the cometary tail divided by the transit velocity), and finally $t_0$, the time of the beginning of the transit. With this model of the transit of the exocometary tail, the corresponding decrease in relative flux at the time *t* is given by $\Delta F/F\, (t) = K \times \left( \exp (-\Delta ) - \exp (-\Delta ^\prime )\right)$, where $\Delta =\beta (t-t_0)$ if $t\ge t_0$, $\Delta =0$ if $t\le t_0$, and $\Delta ^\prime =\beta (t-t_0-\Delta t)$ if $t\ge t_0 +\Delta t$, and $\Delta ^\prime =0$ if $t \le t_0 +\Delta t$.

We calculated the correlation of this 1D-model with the observed light curves of $\beta$ Pic and $\alpha$ Pic by varying the value of the transit time $t_0$ and using various plausible values for the $\Delta t$ and $\beta$ parameters characterizing the shapes of the exocomet transit light curves. We used $\beta$ from 2 to 20 $\hbox {days}^{-1}$ and $\Delta t$ from 0.15 to 0.5 days, corresponding to periastron distances ranging from about 0.08 to 0.85 au. The value of *K* in the model can be arbitrarily chosen because it only changes the amplitude of the light variations, hence it has no consequence in the position of the correlation maximum nor on the identification of exocomet transits events.

In the case of $\beta$ Pic the correlation reaches large positive values for some of the values of $t_0$. This behaviour is not observed with the $\alpha$ Pic light curve, showing that the photometric variations with cometary transit shapes are specific to $\beta$ Pic. We check the negative values of the correlation of the model with the $\beta$ Pic light curve itself, and found that they are much less numerous and significantly smaller than the positive values. This confirms that the light curve of $\beta$ Pic shows photometric variations with decrease of the star brightness that are typical of the transits of exocomets and the absence of variations with increase of the star brightness with similar shape and amplitude. We interpret the correlation peaks as the signatures of potential exocomet transits. We keep only the peaks with correlation values that are higher than the maximum value obtained with the $\alpha$ Pic data and the maximum negative value with the $\beta$ Pic data. This is our conservative criterion to consider the observed variations as a detection of an exocomet transit.

We identified a total of 30 significant detections of exocomet transit events. Although this is not surprising, acknowledging the well known ubiquity of comets in that young planetary system, this is the first time such a large number of exocomets are detected in photometry. This allows statistical analysis of their properties. The light curves of these detected exocomet transits are plotted in Extended Data Fig. 4. Their characteristics and the parameters of the best fits with the 1-D model are given in Extended Data Table 2. The last column of the table gives the square root of the $\chi ^2$ improvements of the fits to the light curve between a model with no transit and the 1-D transit model using the best fit parameters. This shows that the identified exocomet transits are all detected at least at 4-$\sigma$ level.

## Size distribution of $\beta$ Pictoris exocomets

### Distribution of absorption depths

**Figure 1:**
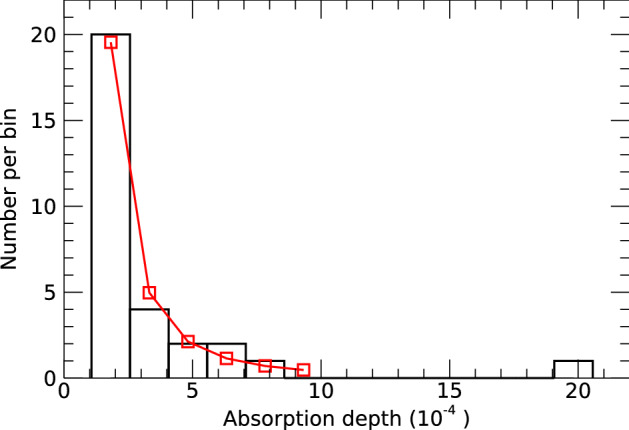
Histogram of the number of exocomet transit events as a function of the absorption depth. The red line shows the fit with a power law function with $\alpha =2.3 \pm 0.4$. The uncertainty on the fitted parameters of the power law function have been evaluated using a Poisson distribution for the number of events in each bin of width $d AD=1.5\cdot 10^{-4}$. The red squares represents the number of expected events in each bin as calculated with the fitted power law.

Our detection of 30 photometric transits of exocomets allows a statistical analysis of their properties. Here we call “absorption depth”, noted *AD*, the decrease in relative flux at the minimum of a transit light curve. The numerical value of the absorption depth is estimated from the best fit with the 1-D model with $AD=K(1-\exp (-\beta \Delta t))$. A plot of the events frequency as a function of the absorption depth shows that there is a steep decrease of the number of events toward the larger absorption depths (Fig. 1). The differential number of transiting exocomet events (*dN*) detected with an observation of duration $\delta t$ as a function of the absorption depth can be fitted by a power law in the form $dN(AD)=N_{0}\cdot (AD/10^{-4})^{-\alpha }\cdot (\delta t/100\, {{\text{days}}})\cdot (dAD/10^{-4})$. Considering 29 events detected in 156 days of observations, that is all the 30 events except the deepest one (see below), we find that $\alpha =2.3 \pm 0.4$ and $N_{0}=33^{+16}_{-11}$, where the uncertainties have been evaluated using a Poisson distribution for the number of events in each bin of width $d AD=1.5\cdot 10^{-4}$.

The deepest transit event^20^ of Julian Day JD = 2457000 + 1486 looks exceptional with an absorption depth of about $20\times 10^{-4}$. This event could be produced by a member of another family of exocomets than the one which produces the 29 other shallower events, as we know from spectroscopic transit observations the presence of several families of $\beta$ Pic comets^5^. Nonetheless, with the distribution derived above, the expected number of events with absorption depths in the range [10–20]$\times 10^{-4}$ in a 156 days observation is $1.2^{+0.6}_{-0.4}$. Even in the range [15–20]$\times 10^{-4}$ the expected number of detections in 156 days is $0.38^{+0.19}_{-0.12}$, corresponding to a probability of 26% to have one single event in this range as observed. Therefore the event of JD = 2457000 + 1486 can simply be a normal event, only the deepest, within the same distribution of the other 29 events. If this event is taken into account, the estimates of the distribution power law indexes $\alpha$ and $\gamma$ change by about 0.1 and 0.2, towards slightly shallower distributions. Nonetheless, to remain conservative, this deepest event is not taken into account in the derivation of the distribution of absorption depths and exocomets sizes considered here.

### Distribution of exocomet sizes

The modeling of the exocomet transit light curves shows that the transit absorption depth, *AD*, is directly proportional to ${\dot{M}}$, the dust evaporation rate from the comet nucleus^17^ . If we assume that the dust production rate is proportional to the comet nucleus area (*i.e.*, Ref.^23^), we have a production rate ${\dot{M}}$ proportional to the squared of the nucleus radius *R*. Finally, with *AD* proportional to $R^2$, we find that the differential number of exocomets as a function of the nucleus size is given by $dN(R) \propto R^{-\gamma } dR$, with $\gamma = 2\alpha -1$.

**Figure 2:**
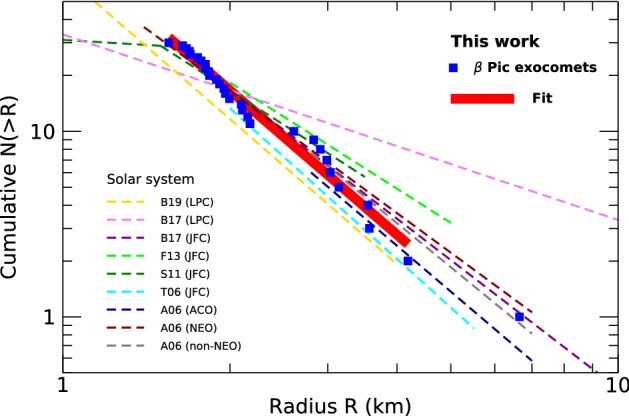
Plot of the cumulative size distribution of the exocomets in $\beta$ Pic. The cumulative size distribution is plotted with blue squares for each exocomet and the corresponding fit excluding the largest comet is plotted with the red thick line. For comparison, published size distributions measured in the Solar system are plotted with thin dashed lines for asteroids in comets orbits (ACO), on near Earth orbits (NEO) and non-near Earth orbits (non-NEO) (A06, Ref.^31^), Jupiter family comets (JFC) (T06, Ref.^26^; S11, Ref.^27^; F13, Ref.^29^; B17, Ref.^30^), and long-period comets (LPC) (B17, Ref.^30^; B19, Ref.^25^). In this plot, the size distributions for the objects in the Solar system have been scaled to have a cumulative number of about 10 objects with radius above 2 kilometers. The radii of the $\beta$ Pic comets have been estimated using the derivation described in the Method section. The conclusion on the similarity of the size distributions in $\beta$ Pic and the Solar system is independent of these estimates.

With the fit to the observed distribution of the absorption depths, we conclude that the differential distribution of the exocomet size must follow a power law with an index $\gamma =3.6 \pm 0.8$. This distribution is notably similar to the size distribution of comets in the Solar system (Fig. 2) and the distribution predicted in Ref.^24^ for a collisionally relaxed population ($\gamma _{{\text{D}}}= 3.5$).

For the plot of the size distribution (Fig. 2), we used the cometary radii estimated following the derivation described in the Method section. The conclusion on the similarity of the size distributions in $\beta$ Pic and the Solar system is independent of these absolute size estimates. Nonetheless, it is remarkable that not only the distribution but also the sizes of the $\beta$ Pic comets nuclei are found to be similar to sizes of the Solar system comets.

### Comparison with Solar system comets

The size distribution of the nucleus of Solar system comets has been estimated for various locations. The size distribution of the Jupiter family comets and Oort cloud comets are found to be similar but not exactly the same.

For the Oort cloud, the size distribution has been estimated using a cometary activity model with a survey simulation and application to 150 long-period comets (LPC) detected over 7 years by the Pan-STARRS1 near-Earth object survey^25^. For objects with diameters above 1 km, the distribution is found to be $\gamma = 3.6\pm 0.4$ (stat.) $\pm 0.7$ (sys.), which is in remarkable agreement with our value for $\beta$ Pic exocomets. For smaller long-period comets, a shallower distribution is found with $\gamma = 0.35$ for diameters between 100 m and 1 km (Ref.^25^). Note that in practice, in a similar manner as we have used the transit absorption depth as a proxy for the size estimate of the $\beta$ Pic comets, the above estimates for the Oort cloud comets have been obtained using the absolute H magnitudes of the nuclei as the proxy for their size.

For the $\beta$ Pic exocomets, the comparison may be more appropriate with the size distribution of the comets in the Jupiter family, which originates from the Kuiper Belt. In Ref.^26^ a catalog of absolute nuclear magnitudes of Jupiter family comets (JFC) has been used to derive a size distribution and to find $\gamma =3.7 \pm 0.3$ for nuclei with radius between 2 and 5.5 kilometers (see Fig. 8 of Ref.^26^). More recent works have provided a shallower distribution : analyzing a large number of optical observations, Ref.^27^ found a lower value with $\gamma =2.9 \pm 0.2$ for nuclei with radius larger than 1.25 kilometers, and $\gamma = 1.2$ for smaller objects. This last result is consistent with the result described in Ref.^28^, where images of Jupiter family comets obtained with the Hubble space telescope and the Keck telescopes have been analyzed. With a model fit to the observations, it is concluded that the intrinsic size distribution of comets in the Jupiter family is consistent with a $\gamma = 3.5$ power-law but truncated at small nucleus radii below 2.0 kilometers. In Ref.^29^ a similar distribution is obtained with $\gamma = 2.9$ for radius between 2 and 5 kilometers, interpreting the distribution shallower than the canonical Dohnanyi’s size distribution $\gamma _{{\text{D}}} = 3.5$ (Ref.^24^) as due to fragmentation of the JFC objects. In Ref.^30^ the measured $\gamma = 3.3 \pm 0.2$ for Jupiter family comets of radius between 2 and 10 kilometers is to be compared to the $\gamma =2.0 \pm 0.1$ for long-period comets between 1 and 20 kilometers in radius.

Finally, in the Solar system the size distribution of extinct or dormant comets can be determined through the population of asteroids in comet orbits (ACO). Assuming that the exhaustion processes have no major impact on the size distribution, the observed distribution for these asteroids can be considered as a good proxy for the distribution for the parent comets. For these objects, Ref.^31^ found $\gamma =3.55 \pm 0.04$ for the full sample with radius between 2.8 and 7 kilometers, $\gamma =3.2 \pm 0.04$ for near Earth objects (NEO) with radius down to 1.4 kilometers and $\gamma =3.45 \pm 0.04$ for non-near Earth objects (non-NEO) with radius between 2.8 and 7 kilometers.

Taken all together these estimates for the Solar system comets are in general agreement with the value that we obtained for the $\beta$ Pic comets, with some slightly shallower distributions in some cases (Fig. 2). This points toward the importance of collisional fragmentation in shaping the size distribution of the exocomets in the younger $\beta$ Pic planetary system.

## Discussion

The measured absorption depth distribution is the result of the distribution of several parameters for each individual comet, *e.g.*, the orbital parameters, cometary activity, composition, size, etc. Here, following the result of numerical simulations^17,18^, we assumed that the absorption depth distribution is mainly dominated by the distribution of the exocomet intrinsic dust production rate and hence their size. In other terms, although other parameters play a role for each individual comet, their diversities are expected to have a lower impact on the observed transit absorption depth than the size. In support of that idea, in spectroscopy it is observed that the $\beta$ Pic transiting comets present similar orbital characteristics, which allows the classification in two different families^5^. With similar orbits, different transiting exocomets have different dust tails mainly because of different dust production rate, and hence because of different size nuclei.

The observed distribution of exocomets in the young planetary system of $\beta$ Pic is strikingly similar to the distribution observed in the Solar system. This distribution seems to be ubiquitous and is also consistent with the canonical Dohnanyi’s size distribution^24^ ($\gamma _{{\text{D}}} = 3.5$), which corresponds to the size distribution of a collisionally relaxed population (see discussion in Ref.^32^). This indicates that the collisional process with fragmentation cascades is likely one of the dominant processes that shape the population of kilometer-sized bodies in the $\beta$ Pic planetary systems.

## Methods

### TESS observations

$\beta$ Pictoris has been observed by TESS at 2-minute cadence several times from October 2018 to February 2021. The available data-set covers a total of 156 days of observations in the optical domain, divided into 14 epochs of about 12 days each. The raw data shows a flux dispersion of about $10^{-3}$, which is mainly due to $\delta$ Scuti pulsations in the stellar atmosphere.

The data from 19 October 2018 to 1 February 2019 have already been analysed^20^. Three photometric events have been identified and attributed to the transits of three different exocomets, with one spectacular transit at Julian Day (JD) equals to (2457000+1486).

In addition to these pioneering observations, $\beta$ Pic has been observed from 20 November 2020 to 8 February 2021. For the observations of Sectors 4 to 7 and Sectors 31 to 34 (Extended Data Table 1), the TESS data products were obtained from the Mikulski Archive for Space Telescopes (MAST). We used the Presearch Data Conditioning (PDC) flux time series^33–35^ processed by the TESS Science Processing Operations Center (SPOC) pipeline version 5.0.23-20210212^36,37^, with the spacecraft cosmic ray mitigation algorithm enabled.

### Cleaning the light curves

#### The $\delta$ Scuti variations

$\beta$ Pictoris is prone to $\delta$ Scuti type photometric variations^38–40^. These variations have a dominant frequency of $47.44\,\hbox {d}^{-1}$ (corresponding to a period of about 0.5 hours), with an amplitude of up to $4\times 10^{-3}$ (Extended Data Fig. 1). These variations are superimposed to the photometric signatures of exocomet transits and must be corrected before searching for these exocomets.

Each of the 14 epochs of continuous observations (Extended Data Table 1) have been reduced separately; for each of them we extracted the set of frequencies and amplitudes of the pulsations using the Period04 software as previously done in Ref.^20^. The Period04 software performs a Fourier transform on the data, giving the frequencies, amplitude, phase and signal to noise ratio of each harmonic in the time series. We conducted the frequency search between 15 and $100\,\hbox {day}^{-1}$, to avoid removing slow variations that might be caused by a cometary transit. For each iteration of the software, the highest-amplitude frequency within the search range is selected, and added to a multi-sine model. This model is then optimized over amplitude and frequency of each harmonic, then removed from the original signal. For each iteration, the software computes the signal to noise ratio of the main remaining frequencies. The exit condition of the loop was chosen when the main frequency’s signal to noise ratio went under 4. We considered that below this limit, all that is left is noise.

The process has been applied for each of the 14 blocks of continuous observations, leading to an average number of 43 different pulsations for each block. After identifying all these pulsations, we subtracted them from the photometric measurements. The resulting residuals were then rebinned from an initial time sampling of 120 seconds to a time sampling of 1800 seconds.

To validate the result, we checked that the three exocomet transits already identified in Ref.^20^ are clearly visible in the data set cleaned from the $\delta$ Scuti variations by this procedure (Extended Data Fig. 2). The data are clean enough that new potential exocomet transits are suspected from the resulting light curve.

#### Remaining slow variations

After removal of the $\delta$ Scuti variations, the resulting light curve still shows slow variations. These variations can have various origins (residuals of the $\delta$ Scuti variations that were not properly eliminated, systematic correlated noise, instrumental effects, etc.). Whatever the origin of these slow variations, astrophysical or instrumental, they need to be cleaned before searching for exocomet transits. To do so, for each epoch of continuous observations we searched for a smooth function modelling these variations. First we rebinned the time series at a 1-day-long interval, in order to “protect” the shorter dimmings (such as comets), excluded the 1-day measurements where the deepest exocometary transits are already clearly identified (by using the detection procedure described in the main text), then we interpolated a function with a cubic spline to model the generic form of the time series. Finally, we normalized our time series with this model. The result is a flattened light curve, which can be directly used to search for shallow dip events due to exocomet transits. The procedure is illustrated in Extended Data Fig. 3 where a potential second shallower exocomet transit closely follows another deep exocomet transit event.

### Comet’s nuclei radius

The conclusion on the size distribution given in the main text does not require to estimate the true size of the comet’s nuclei. It only relies on the assumption that the absorption depth is a good proxy for the dust production rate, which is supported by numerical simulations of exocomets light curves^18^, and that the production rate is proportional to the area of the comet’s nucleus, which is consistent with Solar system comets’ models and observations.

Nonetheless, using these simulations and Solar system observations, we can make a step further and derive the typical sizes of the comets detected in the $\beta$ Pic TESS light curve. From a newly calculated library of exocomet transit light curves similar to the one of Ref.^18^, we derive a typical scaling law for the absorption depth *AD*, which is

$$\begin{aligned} AD\simeq 5\cdot 10^{-5}\left( \frac{{\dot{M_{1\,{{\text{au}}}}}}}{10^5 kg\, s^{-1}} \right) \left( \frac{q}{1\,au}\right) ^{-1/2} \left( \frac{M_*}{M_\odot }\right) , \end{aligned}$$

where $\dot{M_{1\,{{\text{au}}}}}$ is the dust production rate of the comet when it is at 1 au from the star, *q* is the orbital periastron distance and $M_*$ is the mass of the star. Because the transiting comet is seen from a distance that is extremely large compared to the star-comet distance, for a given cometary tail size, thickness and impact parameter, the percentage of the stellar disk covered by the tail and therefore the absorption depth do not depend on the comet distance to the star. The above estimate of the absorption depth is valid over a wide range of impact parameter and wide range of longitude of perisastron of the comet’s orbit, $\omega$.

The periastron distance of the detected comets can be estimated using the transit time $\Delta t$. We have $\Delta t = L_{chord}/v_{{\text{transit}}}$, where $L_{chord}$ is the length of the transit chord of the planetary trajectory on the stellar disk that is crossed by the planet at the velocity $v_{{\text{transit}}}$. The chord length depends on the transit impact parameter : it is twice the planet radius for a central transit with a zero impact parameter and it is zero for a tangential transit with an impact parameter equals to the stellar radius. Here we used the mean chord length obtained assuming a random uniform distribution of the impact parameter from zero to one stellar radius, that is $\overline{L_{chord}}=\pi R_{*}/2$. The transit velocity is given by $v_{{\text{transit}}}=\sqrt{GM_* /2q}(\cos \omega +1)\sim \sqrt{GM_*/q}$. With a $\beta$ Pic radius of $R_*=1.7 R_{\odot }$ (Ref.^41^) and a mass of $M_*=1.75 M_{\odot }$, we find $\Delta t \simeq 13 (\sqrt{q/1\,au}$) hours. The best fits values of $\Delta t$ correspond to distances ranging from 0.03 to 1.3 au, in good agreement for the distances expected for the comet evaporation. The mean value of the estimated periastron distances is about 0.18 au. Using this mean distance, we obtain the following relationship for the observed absorption depth and the dust production rate :

$$\begin{aligned} AD\simeq 2\cdot 10^{-4} \left( \frac{{{\dot{M_{1\,{{\text{au}}}}}}}}{10^5 kg\, s^{-1}}\right) . \end{aligned}$$

Finally, the relation between the evaporation rate and the comet’s nucleus size can be derived by scaling the observation of the Hale-Bopp comet. Using a radius of about 30 kilometers^23,42,43^ and a dust production rate of $2\cdot 10^6\,\hbox {kg}\,\hbox {s}^{-1}$ at 1 au (Ref.^23^) for this well-observed dusty comet, we find $\dot{M_{1\,{{\text{au}}}}}\simeq 2\cdot 10^6\,\hbox {kg}\,\hbox {s}^{-1}\, (R/30\,\hbox {km})^2(L_*/L_{\odot })$. With a $\beta$ Pic luminosity of $8.7L_{\odot }$, we find

$$\begin{aligned} R\simeq 7.2 \, {{\text{km}}} \left( \frac{\dot{M_{1\,{{\text{au}}}}}}{10^6\,{{\text{kg}}} \, {{\text{s}}}^{-1}}\right) ^{1/2}. \end{aligned}$$

All together, we conclude that the radius of the $\beta$ Pic comets nuclei can be estimated using the photometric transit absorption depth with

$$\begin{aligned} R\simeq 1.5 {{\text{km}}} \sqrt{AD/10^{-4}}. \end{aligned}$$

Using this relationship, we derive a size of 1.5 km for the smallest detected comets ($AD\simeq 10^{-4}$), and 6.7 km for the largest comet ($AD\simeq 20\cdot 10^{-4}$). These sizes are remarkably similar to the sizes of comets in the Solar system.

## Supplementary Information

Supplementary Information 1.

## Data Availability

The observational data used in this work are publicly available in the Mikulski Archive for Space Telescope (MAST). The data in the tables and the final cleaned light curves are publicly available on GitHub at https://github.com/lecaveli/BetaPic_TESS.

## References

[1] Vidal-Madjar A, Hobbs LM, Ferlet R, Gry C, Albert CE (1986). The circumstellar gas cloud around Beta Pictoris. II. Astron. Astrophys..

[2] Ferlet R, Hobbs LM, Vidal-Madjar A (1987). The beta Pictoris circumstellar disk. V. Time variations of the Ca II-K line. Astron. Astrophys..

[3] Beust H, Lagrange-Henri AM, Vidal-Madjar A, Ferlet R (1990). The beta Pictoris circumstellar disk. X. Numerical simulations of infalling evaporating bodies. Astron. Astrophys..

[4] Vidal-Madjar A (1994). HST-GHRS observations of $$\beta$$ Pictoris: Additional evidence for infalling comets. Astron. Astrophys..

[5] Kiefer F (2014). Two families of exocomets in the $$\beta$$ Pictoris system. Nature.

[6] Strøm PA (2020). Exocomets from a solar system perspective. Publ. Astron. Soc. Pac..

[7] Miret-Roig N (2020). Dynamical traceback age of the $$\beta$$ Pictoris moving group. Astron. Astrophys..

[8] Smith BA, Terrile RJ (1984). A circumstellar disk around $$\beta$$ Pictoris. Science.

[9] Kalas P, Larwood J, Smith BA, Schultz A (2000). Rings in the Planetesimal Disk of $$\beta$$ Pictoris. Astrophys. J. Lett..

[10] Roberge A, Feldman PD, Weinberger AJ, Deleuil M, Bouret J-C (2006). Stabilization of the disk around $$\beta$$Pictoris by extremely carbon-rich gas. Nature.

[11] Apai D (2015). The inner disk structure, disk-planet interactions, and temporal evolution in the $$\beta$$ Pictoris system: A two-epoch HST/STIS coronagraphic study. Astrophys. J..

[12] Brandeker A (2016). Herschel detects oxygen in the $$\beta$$ Pictoris debris disk. Astron. Astrophys..

[13] Lagrange AM (2010). A giant planet imaged in the disk of the young star $$\beta$$ Pictoris. Science.

[14] Snellen IAG, Brown AGA (2018). The mass of the young planet Beta Pictoris b through the astrometric motion of its host star. Nat. Astron..

[15] Lagrange AM (2019). Evidence for an additional planet in the $$\beta$$ Pictoris system. Nat. Astron..

[16] Lacour, S. *et al.* The mass of Beta Pictoris c from Beta Pictoris b orbital motion. *arXiv e-prints* arXiv:2109.10671 (2021).

[17] Lecavelier des Etangs A, Vidal-Madjar A, Ferlet R (1999). Photometric stellar variation due to extra-solar comets. Astron. Astrophys..

[18] Lecavelier des Etangs A (1999). A library of stellar light variations due to extra-solar comets. Astron. Astrophys. Suppl. Ser..

[19] Rappaport S (2018). Likely transiting exocomets detected by Kepler. Mon. Not. R. Astron. Soc..

[20] Zieba S, Zwintz K, Kenworthy MA, Kennedy GM (2019). Transiting exocomets detected in broadband light by TESS in the $$\beta$$ Pictoris system. Astron. Astrophys..

[21] Ricker GR (2015). Transiting Exoplanet Survey Satellite (TESS). J. Astron. Telesc. Instrum. Syst..

[22] Lecavelier des Etangs A (1993). Observation of the central part of the beta Pictoris disk with an anti-blooming CCD. Astron. Astrophys..

[23] Jewitt D, Matthews H (1999). Particulate mass loss from comet hale-bopp. Astron. J..

[24] Dohnanyi JS (1969). Collisional model of asteroids and their debris. J. Geophys. Res..

[25] Boe B (2019). The orbit and size-frequency distribution of long period comets observed by Pan-STARRS1. Icarus.

[26] Tancredi G, Fernández JA, Rickman H, Licandro J (2006). Nuclear magnitudes and the size distribution of Jupiter family comets. Icarus.

[27] Snodgrass C, Fitzsimmons A, Lowry SC, Weissman P (2011). The size distribution of Jupiter Family comet nuclei. Mon. Not. R. Astron. Soc..

[28] Meech KJ, Hainaut OR, Marsden BG (2004). Comet nucleus size distributions from HST and Keck telescopes. Icarus.

[29] Fernández YR (2013). Thermal properties, sizes, and size distribution of Jupiter-family cometary nuclei. Icarus.

[30] Bauer JM (2017). Debiasing the NEOWISE cryogenic mission comet populations. Astron. J..

[31] Alvarez-Candal A, Licandro J (2006). The size distribution of asteroids in cometary orbits and related populations. Astron. Astrophys..

[32] O’Brien DP, Greenberg R (2005). The collisional and dynamical evolution of the main-belt and NEA size distributions. Icarus.

[33] Smith JC (2012). Kepler presearch data conditioning II: A Bayesian approach to systematic error correction. Publ. Astron. Soc. Pac..

[34] Stumpe MC (2012). Kepler presearch data conditioning I-Architecture and algorithms for error correction in Kepler light curves. Publ. Astron. Soc. Pac..

[35] Stumpe MC (2014). Multiscale systematic error correction via wavelet-based bandsplitting in Kepler data. Publ. Astron. Soc. Pac..

[36] Jenkins, J. M. *et al.* The TESS science processing operations center. In *Software and Cyberinfrastructure for Astronomy IV*, Vol. 9913 of *Proceedings of the SPIE*, 99133E. 10.1117/12.2233418 (2016).

[37] Caldwell DA (2020). TESS science processing operations center FFI target list products. Res. Notes Am. Astron. Soc..

[38] Koen C (2003). $$\delta$$ Scuti pulsations in $$\beta$$ Pictoris. Mon. Not. R. Astron. Soc..

[39] Mékarnia D (2017). The $$\delta$$ Scuti pulsations of $$\beta$$ Pictoris as observed by ASTEP from Antarctica. Astron. Astrophys..

[40] Zwintz K (2019). Revisiting the pulsational characteristics of the exoplanet host star $$\beta$$ Pictoris. Astron. Astrophys..

[41] Kervella, P. *et al.* VINCI/VLTI Observations of Main Sequence Stars. In *Stars as Suns : Activity, Evolution and Planets*, (Eds. Dupree, A. K. & Benz, A. O. ) Vol. 219, 80 (2004). arXiv:astro-ph/0309784.

[42] Fernández YR (1999). The inner coma and nucleus of comet Hale–Bopp: Results from a stellar occultation. Icarus.

[43] Bair, A. N., Schleicher, D. G. & Farnham, T. The Extremely Active Comet C/Hale-Bopp (1995 O1): Production Rates from Nearly Five Years of Narrowband Photometry. In *AAS/Division for Planetary Sciences Meeting Abstracts #50*, Vol. 50 of *AAS/Division for Planetary Sciences Meeting Abstracts* 210.06 (2018).

