# Supplementary material for: Exocomets size distribution in the β Pictoris planetary system
Source: Sci Rep. 2022 Apr 28;12:5855. doi: 10.1038/s41598-022-09021-2 (PMC9051165; doi:10.1038/s41598-022-09021-2)
Supplement: Supplementary file 1 — Supplementary Information 1. [file 41598_2022_9021_MOESM1_ESM.pdf]

Extended Data Figures

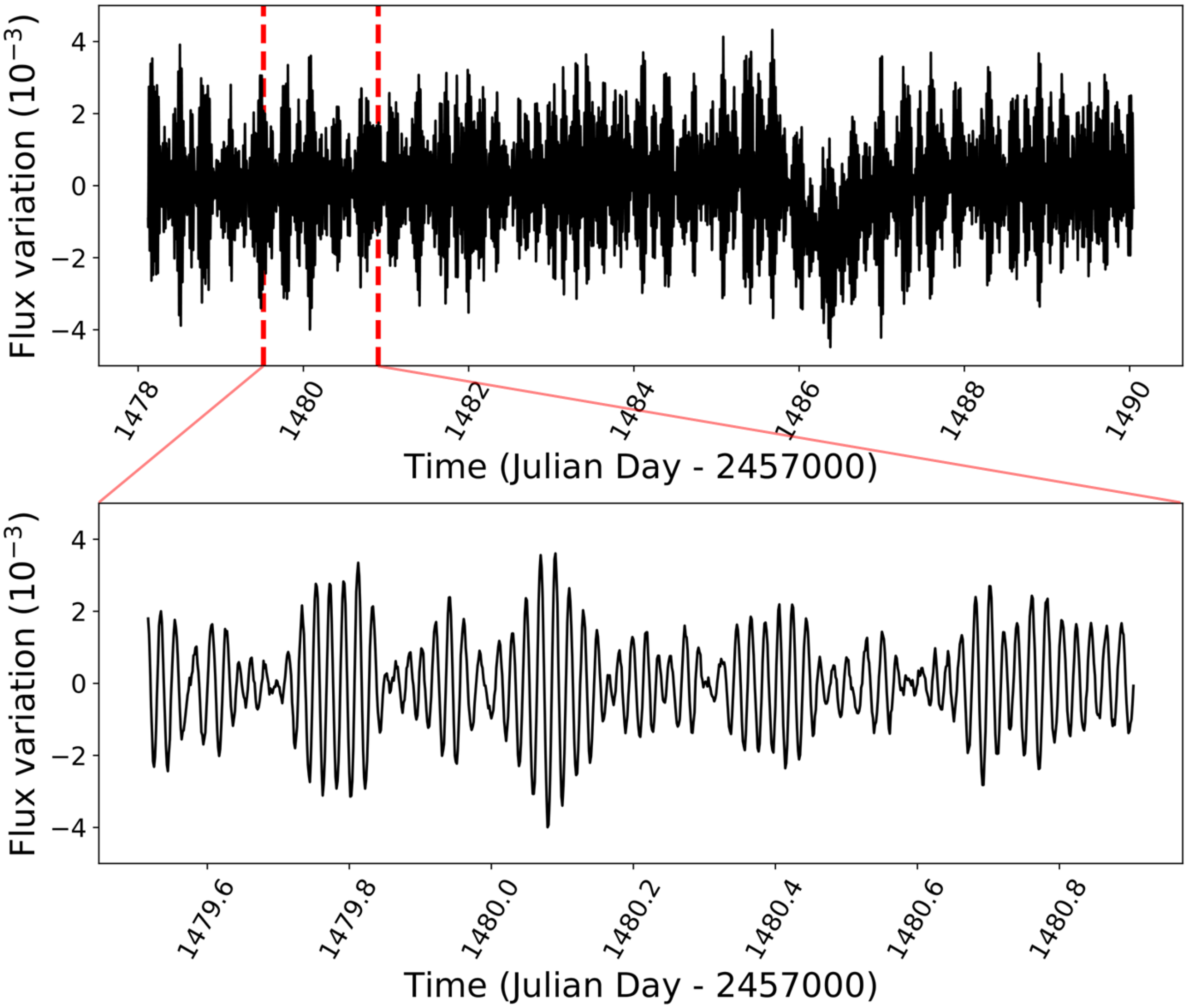

Extended Data Fig. 1. Sample of TESS photometry of β Pic. The light curve is dominated by δ Scuti variations. **The bottom panel shows the variations over about one day and a half with a relative amplitude of up to 4×10^{-3}. At this scale the noise is not clearly visible.**

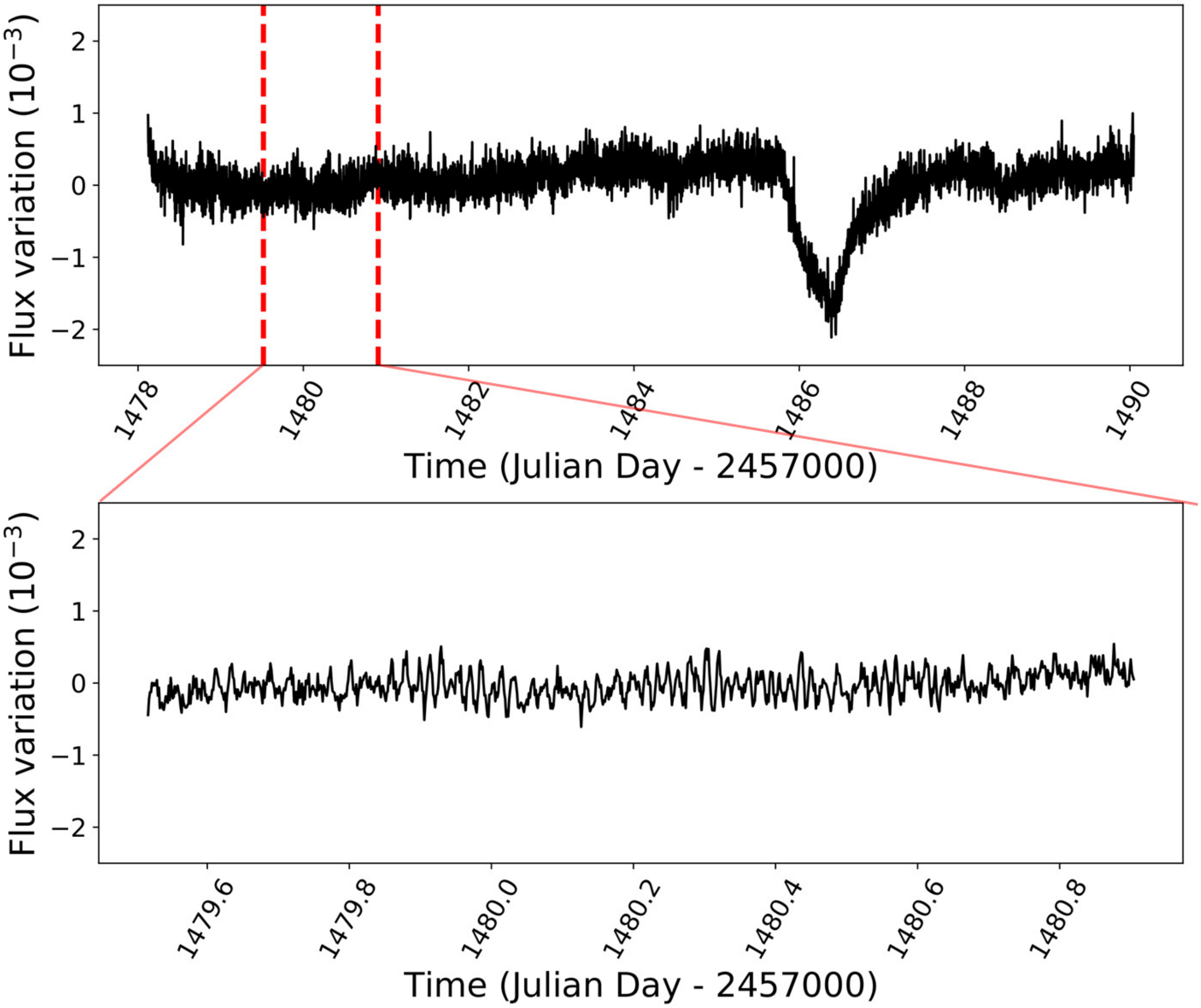

Extended Data Fig. 2. The β Pic TESS light curve after removing the δ Scuti photometric variations. The plot is for the same time interval as in Extended Data Fig. 1. Here the photometric dip at Julian Day 2457000+1486 already identified in ref.[19] is clearly visible.

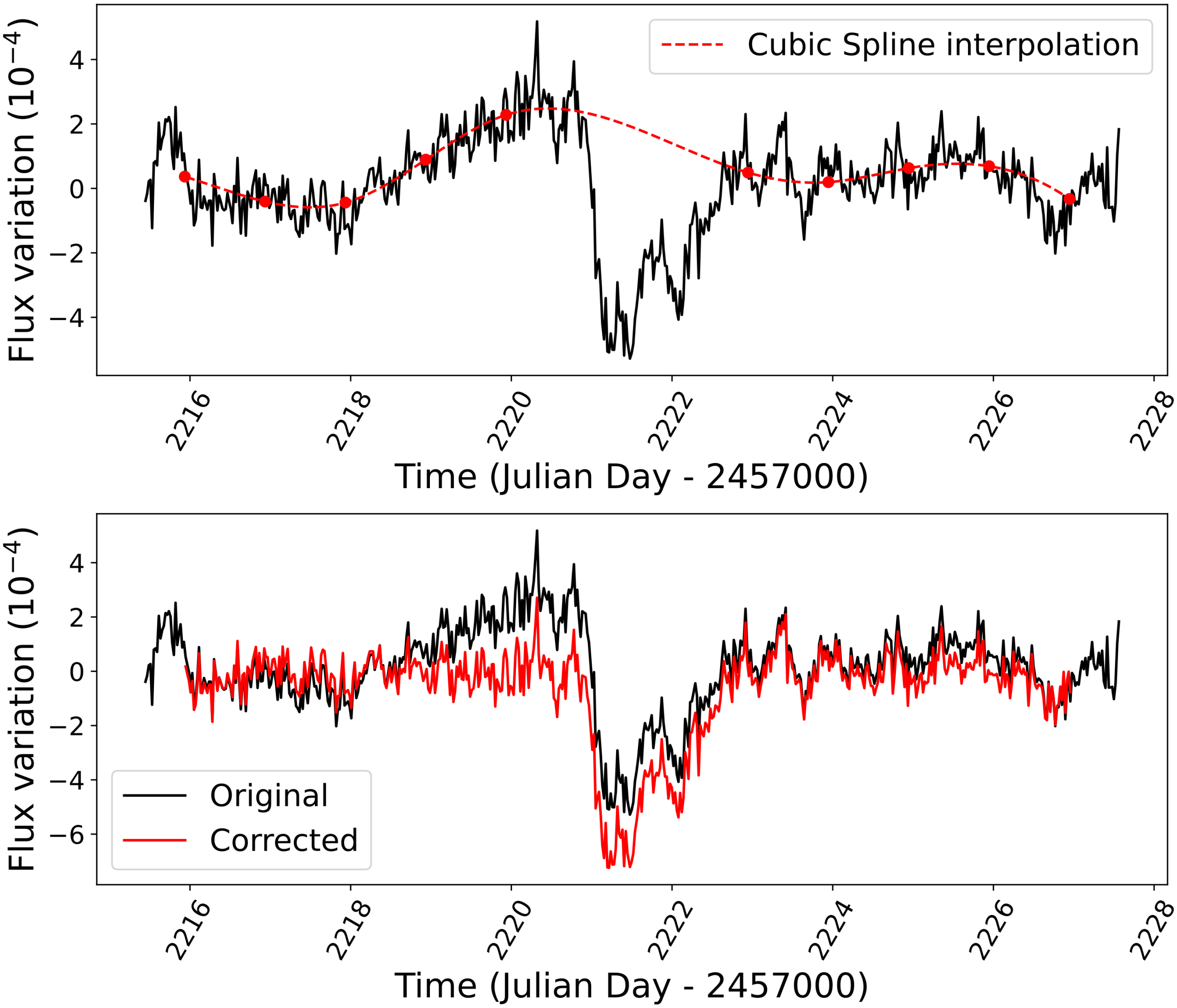

Extended Data Fig. 3. Illustration of the process to remove the slow time scale variations. The top panel shows the data binned with a 1-day interval (red dots) and the cubic spline fitting the rebinned data (red dotted line). The bottom panel shows the original data (black line) and the final data after correction (red line).

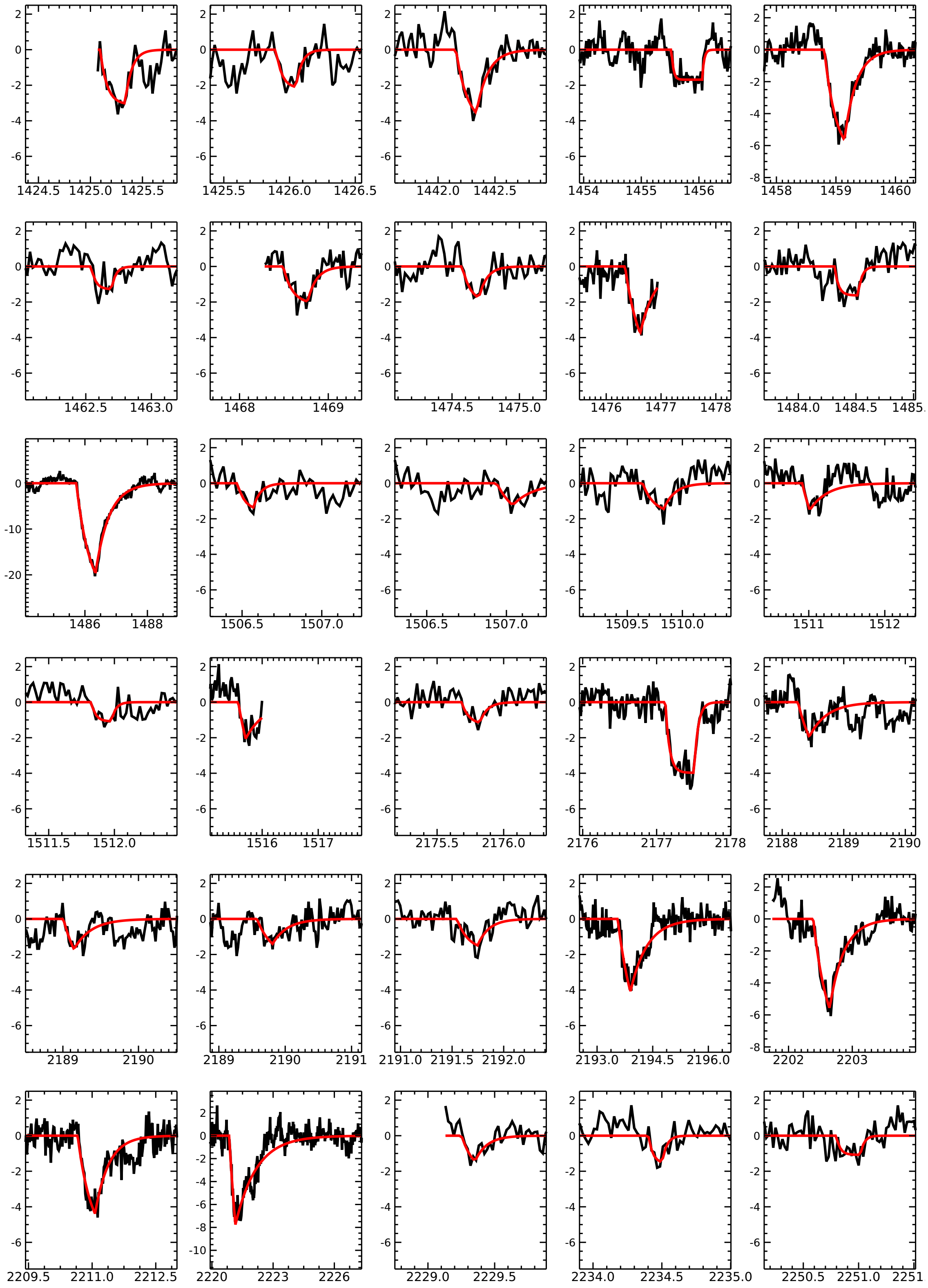

Extended Data Fig. 4. β **Pic photometric variations at the time of the 30 detected transits of exocomets.** The time in the x-axis is given in units of Julian Day-2457000 and the flux variations in the y-axis are relative to the mean flux and given in units of 10^{-4}. The red line represents the best fits with the 1-D model. **The significance level of each exocomet detection is reported in the last column of the Extended Data Table 2.**

Extended Data Tables

Extended Data Table 1. Log of TESS observations. The epochs of the data studied in ref.[19] are noted in bold font.

Date begin (JD-2457000)	Date end (JD-2457000)
**1411.0**	**1418.5**
**1425.1**	**1436.1**
**1438.0**	**1450.1**
**1451.6**	**1463.9**
**1468.3**	**1476.9**
**1478.1**	**1490.0**
**1491.6**	**1503.0**
**1504.7**	**1516.0**
2174.2	2185.7
2187.2	2200.0
2201.7	2213.8
2215.4	2227.5
2229.1	2240.8
2243.0	2253.0

Extended Data Table 2. List of the detected exocomet transits. The second to the fifth columns give the parameters of the best fits with the 1-D model. **The sixth and seventh columns give the corresponding calculated absorption depths and their associated error bars. The last column gives the significance level of each transit detection quantified by the square root of the χ^2 differences between a model with no transit and the 1-D model with the best fit parameters. All the exocomet transits have detection levels above 4-σ (between 4.5-σ and 150-σ, median at 9.1-σ).**

ID	Time t_0	Δt	β	K	Absorption depth		$\sqrt{\Delta\chi^2}$
					value	error	
	(JD-2457000)	(days)	(days^{-1})	(10^{-4})	(10^{-4})		
0	1425.09	0.24	15.44	3.10	3.02	0.26	11.6
1	1425.90	0.15	22.11	2.17	2.10	0.29	6.6
2	1442.16	0.18	9.76	4.32	3.56	0.20	16.2
3	1455.52	0.53	30.00	1.69	1.69	0.15	15.4
4	1458.80	0.34	5.02	6.93	5.65	0.15	36.1
5	1462.54	0.15	30.00	1.29	1.27	0.27	6.2
6	1468.49	0.27	9.85	2.11	1.97	0.21	9.7
7	1474.58	0.12	16.79	2.02	1.77	0.27	6.1
8	1476.34	0.28	3.62	5.94	3.76	0.18	20.0
9	1484.31	0.20	30.00	1.63	1.63	0.29	7.7
10	1485.72	0.62	2.30	25.91	19.63	0.15	148.4
11	1506.47	0.10	21.60	1.54	1.36	0.30	5.0
12	1506.94	0.10	7.59	2.33	1.24	0.30	4.8
13	1509.64	0.19	9.83	1.71	1.45	0.27	7.0
14	1510.91	0.10	4.31	4.32	1.51	0.30	7.0
15	1511.82	0.15	30.00	1.08	1.07	0.25	4.7
16	1515.57	0.13	2.90	6.46	2.06	0.27	10.8
17	2175.68	0.14	17.82	1.26	1.15	0.31	4.5
18	2177.12	0.39	18.45	3.98	3.97	0.15	25.0
19	2188.26	0.18	3.47	4.15	1.95	0.20	12.2
20	2189.01	0.14	4.41	3.69	1.71	0.25	9.1
21	2189.57	0.23	4.47	2.16	1.40	0.15	8.8
22	2191.55	0.20	9.01	1.78	1.49	0.22	7.7
23	2193.56	0.34	2.21	7.69	4.09	0.15	32.3
24	2202.39	0.26	4.45	8.21	5.59	0.17	28.3
25	2210.66	0.41	3.11	6.10	4.39	0.15	27.1
26	2220.84	0.31	0.97	30.12	7.78	0.15	72.0
27	2229.25	0.10	11.22	2.13	1.44	0.28	5.6
28	2234.40	0.10	30.00	1.57	1.50	0.28	5.9
29	2250.80	0.22	28.59	1.08	1.08	0.31	5.3
